# Supplementary material for: The effect of real-time EF automatic tool on cardiac ultrasound performance among medical students
Source: PLoS One. 2024 Mar 28;19(3):e0299461. doi: 10.1371/journal.pone.0299461 (PMC10977790; doi:10.1371/journal.pone.0299461)
Supplement: S1 Appendix — (PDF) [file pone.0299461.s001.pdf]

S1 Appendix – Research Questionnaire:

Participant no.______

Group no.____________

Were you trained using the AI assisted real-time EF tool? (yes/no)

Did you spend time outside class training with the ultrasound devices? (Yes/no)

If yes, how many hours? ______

Gender (male/female): ______

Age (years): _____

Ethnicity: (Jewish/ Bedouin/ non-Bedouin Arab/ other): ___________

Final score in Thoracic Anatomy course: ______

If you have chosen not to participate in this study, mark X in this box: ☐

Thanks for your cooperation!
