# Supplementary material for: The effect of real-time EF automatic tool on cardiac ultrasound performance among medical students
Source: PLoS One. 2024 Mar 28;19(3):e0299461. doi: 10.1371/journal.pone.0299461 (PMC10977790; doi:10.1371/journal.pone.0299461)
Supplement: S2 Appendix — (PDF) [file pone.0299461.s002.pdf]

S2 Appendix - 6-minute exam scoring

View	Criteria	Yes	No
Parasternal Long-Axis View	Correct Alignment	1	0
	Total Endocardial Demarcation	1	0
	Mitral Valve Visualization	1	0
	Aortic Valve Visualization	1	0
Parasternal Short-Axis View- base	Aorta Visualization	1	0
	Tricuspid Valve Visualization	1	0
	Pulmonic Valve Visualization	1	0
	Interatrial Septum Visualization	1	0
Parasternal Short-Axis View Mid-Ventricle	Complete LV Visualization	1	0
	Mitral Valve Visualization	1	0
Parasternal Short-Axis View Apex	Complete LV Visualization	1	0
	Papillary Muscles Visualization	1	0
Apical 4-Chamber View	Open LV	1	0
	RV Visualization	1	0
	Mitral Valve Anatomy	1	0
	Tricuspid Anatomy	1	0
	Open Atrium	1	0
Apical 5-Chamber View	Open LV	1	0
	RV Visualization	1	0
	Mitral Valve Anatomy	1	0
	Tricuspid Anatomy	1	0
	Open Atrium	1	0
	Aortic Visualization	1	0
Apical 2-Chamber View	Open LV	1	0
	Mitral Valve Anatomy	1	0
	Open LA	1	0
Subcostal View	Open RV	1	0
	Pericardial Demarcation	1	0
	Interatrial Septal Visualization	1	0

IVC	IVC Visualization	1	0
	RA Visualization	1	0
Please note that the “yes” and “no” values represent whether the specific criteria are met for each view.<br>Abbreviations: LV- left ventricle; LA – left atrium; RV right ventricle; IVC – inferior vena cava.			
